# Editorial: Chemically modified proteins and oligopeptides: A toolbox for therapeutics, diagnostics, and analytics

**DOI:** 10.3389/fchem.2022.1054229

**Published:** 2022-10-24

**Authors:** Sara Pellegrino, Luca Ronda

**Affiliations:** ^1^ Dipartimento di Scienze Farmaceutiche, Università degli Studi di Milano, Milan, Italy; ^2^ Dipartimento di Medicina e Chirurgia, Università degli Studi di Parma, Parma, Italy; ^3^ IBF-CNR, Pisa, Italy

**Keywords:** protein chemistry, peptides, PEGylation chemistry, glycosylation, RiPPs, radiolabeled peptides, antibody drug conjugates (ADCs)

This Research Topic includes studies that explore chemical modifications of proteins and peptides. Protein chemistry is inspired by Nature, with posttranslational modifications (PTMs) being the key step to obtaining different protein adducts, leading to high cellular function variability.

Ribosomally synthesized and posttranslationally modified peptides (RiPPs) are an example of natural products that have an antimicrobial effect. In this Research Topic, the study by Benjdia and Berteau focuses on understanding the radical S-adenosyl-L-methionine (SAM) enzymes involved in RiPP biosynthesis, in particular, those bringing the formation of thioether bond, epimerization, carbon–carbon, and carbon–oxygen bonds. This class of enzymes, which share common structural features and mechanisms of catalysis, are interesting subjects for the development of novel antimicrobials. Deepening the structural and functional information on SAM will provide information on interaction with the substrate and hence help to engineer novel RiPPs and open novel opportunities for the development of innovative peptide-based antibiotics.

In recent years, understanding of PTMs has also developed in terms of immunopeptidomics. MS and bioinformatic studies have revealed that PTM peptides form 12–25% of the immunopeptidome. PTMs are important in the development of autoimmune diseases, such as systemic lupus erythematosus, and PTM epitopes were identified in several cancers. Sandalova et al. reported recent findings on PTMs epitopes and how they are presented in the Major Histocompatibility Complex (MHC), focusing on glycosylation and phosphorylation.

The detection of PTMs is fundamental in understanding both physiological and pathological conditions, with chemical proteomics representing a new strategy for the total comprehension of PTM events. Emenike et al. describe recent attempts in the development of new chemical methods for labeling different PTMs, with a particular focus on covalent methods. This chemical strategy allows for detecting modifications on low-abundant proteolytic peptide fragments, protein domains, and intact proteins.

Mimicking Nature, the chemical modification of peptides and proteins is a necessary step to making them suitable for use as therapeutics. They have a complex structure and issues such as solubility, stability, and bioavailability have to be addressed. The limits of biologics have been addressed through the introduction of natural and non-natural modifications. Glycosylation, which naturally occurs, confers considerable physiological and biological effects *in vivo*, improving stability and solubility and affecting cell growth, cell-cell adhesion, or cell-pathogen interactions, and hence is used to improve the pharmacokinetics of modified proteins and peptides without causing immunogenicity. Chandrashekar et al. reviewed the most relevant approaches to chemical glycosylation methods, to obtain homogeneous glycopeptides, mainly focusing on the peptides used in the treatment of diabetes.

Chemical modification of peptides is also important in diagnostic, being peptide-based radiopharmaceuticals promising molecular imaging probes. Radiolabeled peptides are, indeed, quickly eliminated from blood and tissues and produce high contrast for imaging applications. Li et al. reviewed the recent preclinical and clinical literature on radiolabeled peptides for prostate cancer diagnosis and therapy.

Besides the chemical nature of the modification, residue reaction specificity is a relevant aspect of protein and peptide chemical modification that is frequently overlooked. This aspect becomes relevant when the modification of a particular protein site alters its functionality. Considering that the most common chemical approaches do not generate a homogenous product, at least part of the modification may affect the desired protein function. This theme is analyzed by Martinez et al. with a particular focus on antibody-drug conjugates (ADCs). Different methodologies for site-specific modification of typically modified aminoacids (lysine, tryptophan, cysteine, histidine, and tyrosine) were reported, pointing out that the protein target is the main player rather than the chemistry used in the obtainment of a homogenous modification. Moreover, the authors highlight how possible loss of function of some specific sites can reveal unknown functions on these residues on the target and suggest possible druggable pockets.

In a protein chemical modification protocol an efficient, homogeneous modification compatible with protein stability, is not the only objective: when proteins are developed to be used *in vivo*, the stability of the chemical modifications upon administration has to be guaranteed. The role of different conjugation chemistries in creating stable binding on cysteine residues in conditions similar to the plasma has been analyzed by Cooper et al. They discuss a recently engineered hemoglobin with a single reactive cysteine residue on the surface to create a single PEGylation site. This hemoglobin mutant was PEGylated with maleimide-PEG, which is known to undergo deconjugation *via* retro-Michael reactions and cross-conjugation to endogenous thiol species *in vivo*, and mono-sulfone-PEG, that is less susceptible to deconjugation. They demonstrate that although maleimide-PEGylation is certainly stable enough for acute therapeutic use, for pharmaceuticals intended for longer vascular retention (weeks or months), reagents such as mono-sulfone-PEG may be more appropriate.

